# *C9orf72* Toxic Species Affect ArfGAP-1 Function

**DOI:** 10.3390/cells12152007

**Published:** 2023-08-05

**Authors:** Simona Rossi, Michela Di Salvio, Marilisa Balì, Assia De Simone, Savina Apolloni, Nadia D’Ambrosi, Ivan Arisi, Francesca Cipressa, Mauro Cozzolino, Gianluca Cestra

**Affiliations:** 1 Institute of Translational Pharmacology (IFT), National Research Council (CNR), 00133 Rome, Italy; simona.rossi@ift.cnr.it (S.R.); i.arisi@ebri.it (I.A.); 2 Fondazione Santa Lucia IRCCS, c/o CERC, 00143 Rome, Italy; nadia.dambrosi@uniroma2.it; 3 Institute of Molecular Biology and Pathology (IBPM), National Research Council (CNR), 00185 Rome, Italy; michela.disalvio@cnr.it; 4 Department of Biology and Biotechnology, University of Rome “La Sapienza”, 00185 Rome, Italy; marili90@hotmail.it (M.B.); desimone.1688910@studenti.uniroma1.it (A.D.S.); 5 Department of Biology, University of Rome “Tor Vergata”, 00133 Rome, Italy; savina.apolloni@uniroma2.it; 6 European Brain Research Institute “Rita Levi-Montalcini”, 00161 Rome, Italy; 7 Department of Ecological and Biological Science, University of Tuscia, 01100 Viterbo, Italy; francesca.cipressa@unitus.it

**Keywords:** amyotrophic lateral sclerosis, *C9orf72*, *Drosophila melanogaster*, Golgi-to-ER trafficking, ArfGAP-1

## Abstract

Compelling evidence indicates that defects in nucleocytoplasmic transport contribute to the pathogenesis of amyotrophic lateral sclerosis (ALS). In particular, hexanucleotide (G4C2) repeat expansions in *C9orf72*, the most common cause of genetic ALS, have a widespread impact on the transport machinery that regulates the nucleocytoplasmic distribution of proteins and RNAs. We previously reported that the expression of G4C2 hexanucleotide repeats in cultured human and mouse cells caused a marked accumulation of poly(A) mRNAs in the cell nuclei. To further characterize the process, we set out to systematically identify the specific mRNAs that are altered in their nucleocytoplasmic distribution in the presence of *C9orf72*-ALS RNA repeats. Interestingly, pathway analysis showed that the mRNAs involved in membrane trafficking are particularly enriched among the identified mRNAs. Most importantly, functional studies in cultured cells and *Drosophila* indicated that *C9orf72* toxic species affect the membrane trafficking route regulated by ADP-Ribosylation Factor 1 GTPase Activating Protein (ArfGAP-1), which exerts its GTPase-activating function on the small GTPase ADP-ribosylation factor 1 to dissociate coat proteins from Golgi-derived vesicles. We demonstrate that the function of ArfGAP-1 is specifically affected by expanded *C9orf72* RNA repeats, as well as by *C9orf72*-related dipeptide repeat proteins (C9-DPRs), indicating the retrograde Golgi-to-ER vesicle-mediated transport as a target of *C9orf72* toxicity.

## 1. Introduction

The expansion of a GGGGCC (G4C2) repeat in the first intron of the *C9orf72* gene is the most common genetic cause of amyotrophic lateral sclerosis (ALS) and frontotemporal dementia (FTD) [1,2]. The pathogenicity of *C9orf72* mutations might arise from the decreased expression of *C9orf72*-encoded protein isoforms, the accumulation of intronic RNAs containing expanded repeats, and/or the formation of dipeptide repeat proteins (C9-DPRs) that are produced by the unconventional, repeat-associated non-ATG (RAN) translation of the intronic expansion. Accordingly, the decreased expression of C9orf72 protein isoforms, the formation of nuclear RNA agglomerates (foci), and the aggregation of C9-DPRs have been recorded in tissues from patients and in cellular and animal systems modeling the disease [3,4], supporting the idea that a combination of these events underlies neuron degeneration in ALS and FTD. Among the numerous mechanisms that have been described as possible contributors to *C9orf72* toxicity, the impairment of nucleocytoplasmic transport (NCT) that occurs in *C9orf72*-ALS has attracted increasing attention [5]. Indeed, it has been shown that *C9orf72*-derived expanded RNAs bind to RanGAP1, a GTPase-activating protein that controls the activity of Ran GTPase, thereby participating in the regulation of NCT [6]. This concept has found a strong confirmation from genetic interaction experiments showing that modifying the expression of key players of NCT, including nuclear pore protein complex-like nucleoporins (Nups) or nuclear transport receptors (NTRs), has strong effects on *C9orf72* toxicity [6]. Interestingly, *C9orf72* toxicity is also affected by RNA nuclear export factors, such as ALYREF and GLE1, suggesting that an impairment of RNA trafficking might be involved [7,8]. Evidence has also been produced indicating that C9-DPRs directly affect NCT by interacting with a number of proteins involved in NCT regulation, including Nups and NTRs [6,9]. Moreover, the formation of stress granules, which is induced via the cytoplasmic accumulation of ALS-related proteins, including *C9orf72*-derived DPRs, causes NCT impairments due to the sequestration of key regulators of this process into stress granules [10,11,12]. Despite these advancements, it remains unclear how defective NCT leads to motor neuron (MN) degeneration.

To begin clarifying this issue, we set out to identify mRNAs whose nucleocytoplasmic localization is specifically affected by *C9orf72* expanded repeats and to verify in vivo the functional significance of this alteration. Interestingly, here we found that the subcellular localization of several mRNAs encoding membrane trafficking proteins is altered in cells expressing *C9orf72* RNA repeats. Beginning with this evidence, we demonstrated that *C9orf72* toxic species directly affect the membrane trafficking route regulated by ADP-Ribosylation Factor 1 GTPase Activating Protein (ArfGAP-1). ArfGAP-1 is a GTPase-activating protein recruited on the Golgi membrane to promote the hydrolysis of GTP on small GTPase ADP-ribosylation factor 1 (Arf1). Such Arf1 hydrolysis is required to disassemble COPI coat proteins from Golgi-derived vesicles, which is essential for their fusion with target membrane compartments [13,14]. Interestingly, the tridimensional structure of the C9orf72 protein complex was recently resolved via cryo-EM, and it was demonstrated that the C9orf72-SMCR8 dimer behaves as a GTPase-activating protein on Arf1 [15,16]. These works support a model in which C9ORF72 haploinsufficiency contributes to motor neuronal dysfunction by hampering GAP activity on Arf1 [15,16]. Accordingly, we demonstrate that ArfGAP-1 function is specifically affected by expanded *C9orf72* RNA repeats, as well as by *C9orf72*-related dipeptide repeat proteins (C9-DPRs), indicating retrograde Golgi-to-ER vesicle-mediated transport as a target of *C9orf72* toxicity.

## 2. Materials and Methods

### 2.1. Cell Culture and Transfection

HeLa cells were grown in DMEM with Glutamax (Corning, Corning, NY, USA) and the addition of 10% fetal bovine serum (FBS, Euroclone, Milan, Italy) at 37 °C and 5% CO_2_. For transient transfection experiments, cells that were 80% confluent were transfected with the appropriate plasmids using Lipofectamine 2000 (Invitrogen, Waltham, MA, USA) and collected after 24 h for subsequent analysis. A pcDNA5-expressing control (G4C2)_2_ or expanded (G4C2)_31_ were constructed as previously described [8]. The pCMV plasmids coding for FLAG-tagged glycine–arginine (GR), proline–arginine (PR) or glycine–alanine (GA) dipeptides were generously supplied by Prof. Angelo Poletti [17].

### 2.2. Subcellular Fractionation for RNA and Protein Extraction

For the extraction of RNA from nuclear and cytoplasmic subcellular fractions, the cells were centrifuged at 600× *g* at 4 °C for 5 min and washed in ice-cold PBS. The pellets were suspended in hypotonic lysis buffer (HLB) containing 10 mM Tris pH 7.5, 3 mM MgCl_2_, 10 mM NaCl, 0.1% NP-40, 10% glycerol, protease inhibitors, and 40 U RNAsin and kept on ice for 10 min. The lysates were centrifuged at 1000× *g* at 4 °C for 3 min. Pellets containing nuclear fractions were washed in ice-cold HLB three times, centrifuged at 300× *g* and 4 °C for 2 min, and lysed in TRIzol reagent (Invitrogen, Waltham, MA, USA), according to the manufacturer’s instructions. Supernatants containing cytoplasmic fractions were cleared via centrifugation at 5000× *g* for 5 min, stored at −20° for at least 1 h, and supplemented with 0.3 M sodium acetate and 2.5 volumes of ethanol (100%). Then, cytoplasmic RNAs were precipitated at 16,000× *g* for 15 min at 4 °C. After washing them with ice-cold 70% ethanol and 0.3 M sodium acetate, semi-dry RNA pellets were lysed in TRIzol (Invitrogen, Waltham, MA, USA), using standard procedures.

For the extraction of proteins from nuclear and cytoplasmic fractions, a similar protocol was used with minor changes. In particular, the cells were lysed in HLB buffer containing protease inhibitors for 10 min in ice. An aliquot of cell lysates was harvested and lysed in Laemmli buffer to obtain the total extract, while the remaining lysates were subjected to centrifugation at 1000× *g* for 3 min at 4 °C. The cytoplasmic fractions were obtained by centrifuging the supernatants at 5000× *g* for 5 min. Pellets containing nuclear fractions were washed and lysed in ice-cold lysis buffer (20 mM Tris pH 7.5, 150 mM KCl, 3 mM MgCl_2_, 10% glycerol, 0.3% NP-40, and protease inhibitors). Extracts from the nuclei were sonicated and centrifuged at 16,000× *g* for 15 min at 4 °C. Both the cytoplasmic and nuclear protein extracts were quantified using a Bradford assay (Bio-Rad Laboratories, Hercules, CA, USA). For Western blot analysis, nuclear, cytoplasmic, and total proteins from the same amounts of cells were resuspended in the same volume of 1X Laemmli buffer and loaded at a volume ratio of 1:1:1 for the following SDS-PAGE procedure. 

### 2.3. Electrophoresis and Western Blot

The proteins were analyzed via SDS-PAGE and transferred onto nitrocellulose membranes (GE Healthcare, Chicago, IL, USA). After blocking in TBS, 0.1% Tween-20 (TBS-T), and 5% non-fat milk, the membranes were kept in TBS-T, 2% non-fat milk, and the following primary antibodies (Santa Cruz Biotechnology, Dallas, TX, USA): goat anti-hnRNP H and rabbit anti-GAPDH (FL-335). After rinsing the membranes with TBS-T solution, TBS-T containing 1% non-fat dry milk and the appropriate peroxidase-conjugated secondary antibody (Bio-Rad) were added to the membranesthat were then developed using the ECL chemiluminescence system (Roche, Basel, Switzerland). Fiji ImageJ (NIH, Bethesda, MD, USA) was used to quantifythe resulting signals 

### 2.4. RT-qPCR

Total RNAs isolated from the cytoplasmic and nuclear fractions were digested using RNAse-free DNase (Promega, Madison, WI, USA), following the manufacturer’s protocol. First, 1 μg of RNA was retrotranscribed using a SensiFAST™ cDNA Synthesis Kit (Meridian Bioscience, Memphis, TN, USA). A real-time PCR (qPCR) was performed using iTaq Universal SYBR Green Supermix (Bio-Rad Laboratories, Hercules, CA, USA), using 350 nM of primers (listed in Supplementary Table S1) and 12.5 ng of cDNA. The CFX Connect System (Bio-Rad Laboratories, Hercules, CA, USA) was used for the qPCR reactions. The “single threshold” mode was used to calculate the Cq values. Relative expression levels were normalized to those of the housekeeping Malat1 and GAPDH for nuclear and cytoplasmic RNAs, respectively.

### 2.5. Library Preparation and RNA Sequencing

IGA Technology Services (Udine, Italy) performed the library preparation and RNAseq, using the “TruSeq Stranded mRNA Sample Prep kit” (Illumina, San Diego, CA, USA). The quality of the RNA samples was tested via an Agilent 2100 Bioanalyzer (Agilent technologies, Santa Clara, CA, USA). The libraries were processed with Illumina cBot for cluster generation on the flowcell according to the manufacturer’s protocols and sequenced on single-end mode on a HiSeq2500 (Illumina, San Diego, CA, USA). Raw data were processed via the CASAVA 1.8.2 version of the Illumina pipeline. In particular, data processing included trimming, which was achieved with ERNE and Cutadapt software (TU Dortmund, Germany) [18], and alignments with STAR on the UCSC mm10 reference genome/transcriptome, using default parameters [19]; transcripts were counted using Stringtie [20], and quality control was carried out using the RSeqQC package [21]. The transcript expression values were aggregated per gene symbol. The FPKM values were used for the following analysis. In the case of genes mapped to different genomic loci, they were aggregated by the average. 

The RNAs prepared from samples in triplicate were analyzed via mRNA sequencing. As a first read-out, we obtained the expression levels of 25,366 genes. Filtering was then performed considering an expression threshold of log_2_ (FPKM) > −1.0 and the coordinate expressions of the target mRNAs in all three biological replicates of the experiment. A final set of about 9000 genes was obtained. As a measure of the nucleo-cytosolic localization of the mRNAs, we calculated the cytosol/nucleus (C/N) ratio of each identified mRNA per sample and then calculated the mean value between the three biological replicates. Then, we computed the ratio between the C/N ratios of cells expressing the expanded repeat and compared these values to control cells (fold change, FC). Genes that were differentially distributed between the two compartments in C9orf72 cells compared to control cells were identified via a *t*-test, setting *p* < 0.05 as threshold. Raw and processed data are accessible at GEO, using the accession number GSE235448.

The bidirectional hierarchical clustering heatmap was generated using FunRich software (version 3.1.3) [22]. Reactome pathway analysis was performed using the Enrichr webtool [23], and graphic displaying was achieved via SRPLOT (https://www.bioinformatics.com.cn/en, accessed on 17 April 2023).

### 2.6. FISH and Immunofluorescence Analysis

Fluorescence in situ hybridization (FISH) was carried out as in [8]. In brief, cell cultures seeded on poly-L-lysine-coated glass coverslips were treated with 4% paraformaldehyde in PBS for 10 min and permeabilized in 70% ethanol. Probe hybridization was performed overnight using 250 ng/mL of Cy3-labeled (C4G2)_4_ in a hybridization buffer containing 30% formamide at 37 °C. After the washing steps were completed, the coverslips were mounted on the slides with the use of a Fluoromount mounting medium (Merck, Darmstadt, Germania) or processed for the immunofluorescence analysis, which was performed as follows. After 30 min in PBS containing 1% BSA, the cells were kept at 37 °C for 1 h with primary antibodies in PBS containing 1% BSA, followed by incubation with the secondary antibodies diluted in the same buffer for 45 min at R.T. 

Immunofluorescence analysis in the absence of FISH analysis was performed similarly except for permeabilization, which was performed using 0.1% Triton X-100 in PBS for 5 min, and blocking, which was performed for 30 min in PBS containing 2% FBS. The cell nuclei were stained with DAPI (1 μg/mL in PBS) for 5 min. The cells were analyzed under a LEICA TCS SP5 confocal microscope, and the images were processed via LAS AF and Adobe Photoshop CC2015 software. The following antibodies were used: mouse anti-ARFGAP-1 (C-4) (Santa Cruz Biotechnology, Dallas, TX, USA), mouse anti-GM130 (BD Transduction Laboratories, Franklin Lakes, NJ, USA), mouse anti-KDEL (10C3), rabbit anti-FLAG (Merck, Darmstadt, Germania), Alexa Fluor 488-conjugated goat anti-mouse IgG (Invitrogen, Waltham, MA, USA), Cy3-conjugated donkey anti-mouse IgG, and Alexa Fluor 488-conjugated donkey anti-rabbit IgG (Jackson ImmunoResearch Laboratories, West Grove, PA, USA). 

### 2.7. Analysis of Golgi and ER Morphology

The fragmentation of the Golgi apparatus was analyzed as previously described [24]. In particular, cells presenting dispersed, punctate structures compared to the typical condensed, perinuclear ribbon-like Golgi staining in control cells were described as “fragmented”. Similarly, the disorganization of the ER was recognized via the disruption of perinuclear staining following an immunofluorescence analysis with an anti-KDEL antibody. The percentage of cells containing a fragmented Golgi apparatus or a disorganized ER was scored from at least 100 cells per group from three independent experiments. 

### 2.8. Drosophila Strain and Procedures

RNAi fly lines targeting ArfGAP-1 gene expression (63645 (y[1] v[1]; P{y[+t7.7] v[+t1.8] = TRiP.HMJ30212}attP40) and 50584 (y[1] sc[*] v[1]; P{y[+t7.7] v[+t1.8] = TRiP.GLC01706}attP2)) and RNAi line to downregulate ERGIC-53 (55657 (y1 sc* v1 sev21; P{TRiP.HMC03809}attP40)) were obtained from the Bloomington Stock Center (http://flybase.bio.indiana.edu/). After a quantitative real-time PCR analysis conducted to examine the RNAi-mediated downregulation of ArfGAP-1 in different fly tissues, we focused our studies on the 63645 fly RNAi line. The Bloomington Stock Center also provided all the GAL4 drivers and the other strains utilized. Drosophila stocks and crosses were maintained on Drosophila standard medium (Nutri-fly Genesee Scientific, El Cajon, CA, USA) at 25 °C, unless otherwise indicated.

### 2.9. Statistical Analysis

The statistical analysis was conducted using an unpaired two-tailed Student’s *t*-test for the comparison between two groups or a one-way ANOVA and Tukey’s test for multiple group comparisons, using GraphPad Prism 6 unless otherwise specified. Values that significantly differed from the relative control with *p* values ≤ 0.05 were considered significant.

## 3. Results

### 3.1. Mapping the Nucleo-Cytoplasmic mRNA Distribution in HeLa Cells Expressing C9orf72 31 Repeats

**Figure 1:**
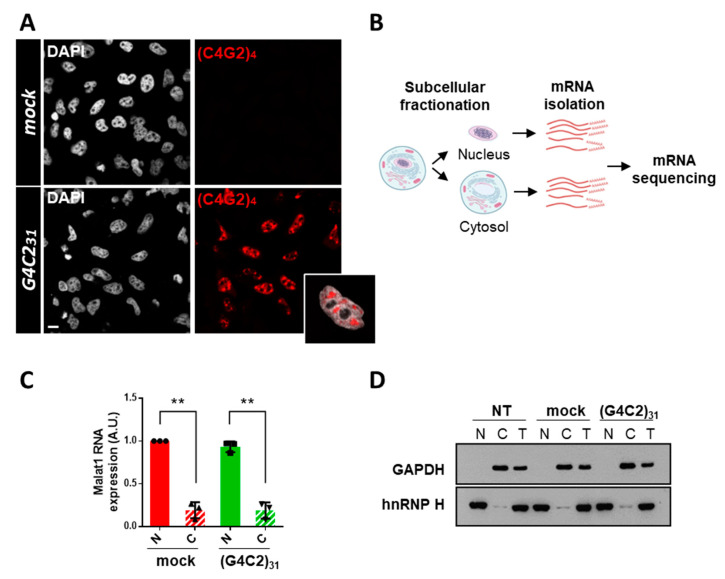
Isolation of nuclear and cytoplasmic fractions from cells expressing expanded G4C2 repeats to profile mRNA localization. (**A**) HeLa cells were transfected with a control empty plasmid (mock) or a plasmid coding for 31 repeats of the hexanucleotide G4C2 (G4C2_31_). After 24 h, cells were subjected to fluorescence in situ hybridization (FISH) with a (C4G2)_4_ RNA probe conjugated to CY3. A high proportion of (G4C2)_31_-transfected cells show nuclear RNA foci, which are absent in control mock-transfected cells. A representative magnification of nuclear RNA foci merged with DAPI is also shown. Scale bar: 10 μm. (**B**) Schematics of the experimental workflow to isolate and profile mRNA distribution between the nuclear and cytosolic compartments. (**C**) RNAs extracted from nuclear (N) and cytosolic (C) fractions of transfected cells as in (**A**) were subjected to qPCR analysis of the nuclear lncRNA Malat1. Gapdh was used as a housekeeping gene. SD was calculated from n = 3 independent experiments. ** *p* < 0.01. (**D**). Equal volume of protein extracts from whole cells (T), nuclear (N), and cytosolic (C) fractions obtained from untransfected (NT) cells or transfected as in (**A**) were loaded on SDS-PAGE gel, in a volume ratio of 1:1:1 and analyzed via Western blot using antibodies that recognize nuclear (hnRNPH) and cytoplasmic (GAPDH) protein markers. A representative image from n = 3 independent experiments is shown.

**Figure 2:**
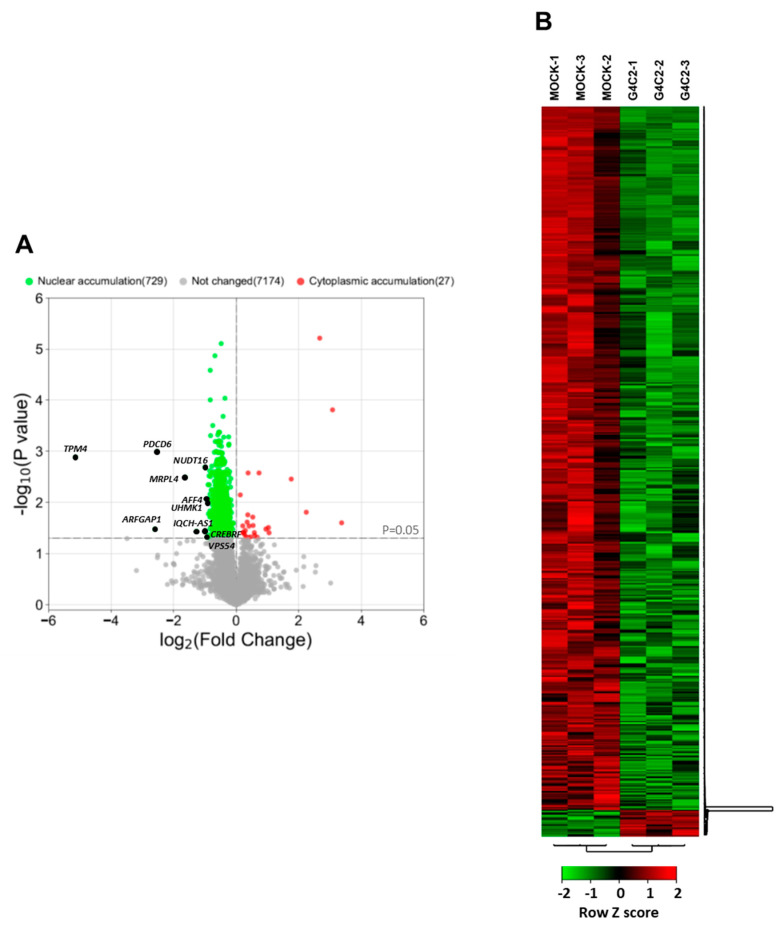
RNAseq profiling of mRNAs from nuclear and cytoplasmic fractions of cells expressing expanded G4C2 repeats. (**A**) Volcano plot shows the intracellular distribution of mRNAs detected in the nuclear and cytoplasmic fractions from G4C2_31_-transfected cells compared to mock-transfected cells (log_2_FC). Statistically significant changes were calculated using an unpaired two-sided *t*-test with a *p*-value cut-off of 0.05 (horizontal grey line). Green and red dots highlight mRNAs accumulated in the nuclear and cytoplasmic fractions, respectively. Nuclear-enriched mRNAs with the top 10 log_2_FC values are indicated with black dots. (**B**) Hierarchical clustering heatmap of differentially distributed RNA with *p*-values < 0.05 of G4C2_31_ transfected cells compared to mock-transfected cells. The color code shows the row z-score, with a green color indicating nuclear RNA accumulation and red color indicating cytoplasmic RNA accumulation.

To extend our previous observations indicating that the nucleo-cytoplasmic distribution of mRNAs is affected by expanded G4C2 repeats [8], we used an RNA-Seq analysis to study the distribution of mRNAs in HeLa cells expressing an expanded (G4C2)_31_ RNA repeat. This cellular approach reproduces ALS-relevant phenotypes such as RNA foci (Figure 1A) and alterations in mRNA trafficking [8]. To this aim, nuclear and cytoplasmic RNAs from mock or *C9orf*72-transfected HeLa cells were extracted (Figure 1B). The efficiency of fractionation was assessed by following the distribution of specific proteins and transcripts in the different fractions via Western blot and qRT-PCR analysis (Figure 1C,D). RNAs prepared from each fraction were analyzed via mRNA sequencing, and the expression levels of 25,366 genes were obtained as a first read-out. After filtering (see the Materials and Methods section), we restricted the dataset to 9000 genes. We calculated the cytoplasm/nucleus (C/N) ratio of each identified RNA per sample and then the mean value between the three biological replicates. Then, we assessed the C/N ratios of mRNAs from cells expressing the expanded repeat and compared them to control cells (fold change, FC). This analysis led to the identification of 756 mRNAs that are differentially distributed between the two compartments in *C9orf72* cells compared to control cells with a *p*-value ≤ 0.05 (Supplementary Table S2). As shown by the volcano plot and heat map, 729 of them are accumulated in the nuclei of *C9orf72* cells (Figure 2A,B). On the contrary, only 27 mRNAs display cytoplasmic accumulation, suggesting that the nuclear retention of mRNAs is a major effect of *C9orf72* expansion.

### 3.2. Pathway Enrichment Analysis of Nuclear-Retained mRNAs Reveals Golgi-to-ER Trafficking as a Major Target of Expanded C9orf72 RNA Repeats

**Figure 3:**
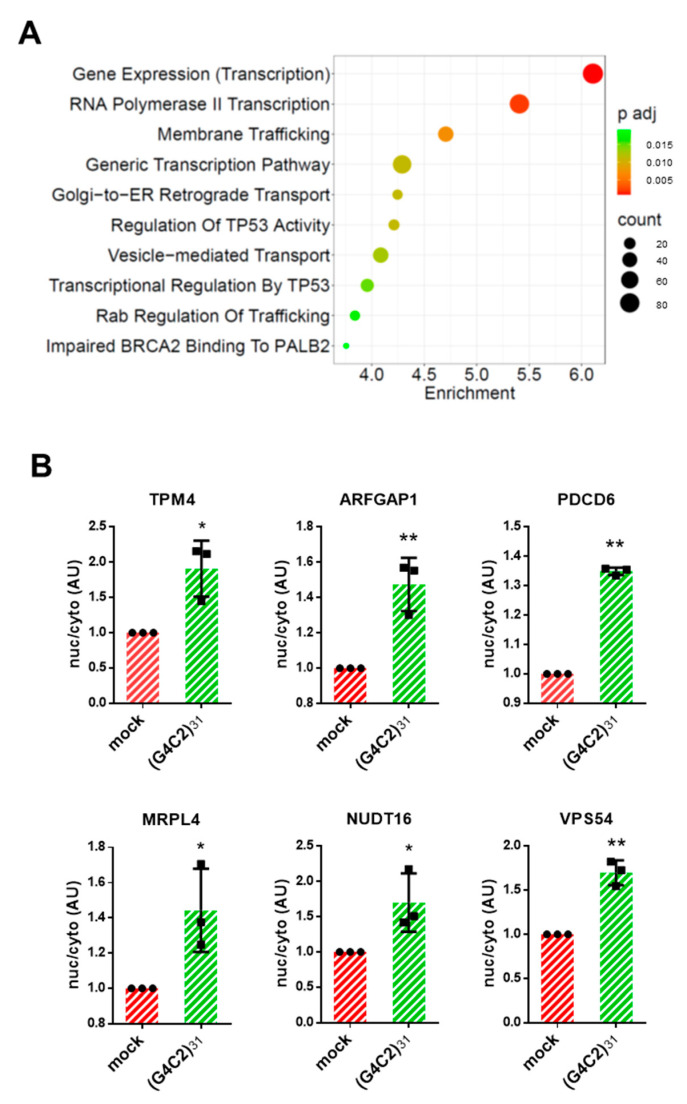
Pathway enrichment analysis of mRNAs retained in the nuclei of cells expressing G4C2 expanded repeats. (**A**) Pathway analysis of nuclear-retained mRNAs in (G4C2)_31_ expressing cells, carried out using Enrichr. The top 10 terms for Reactome pathway analysis are displayed based on decreasing −log_10_ *p*-values (enrichment). The color code shows the adjusted *p*-value, while the sizes of the bubbles represent the number of genes enriching the corresponding annotation (count). (**B**) HeLa cells were transfected with a control plasmid (mock) or a plasmid coding for (G4C2)_31_. Total RNAs were extracted from the purified nuclear and cytosolic fractions and analyzed via RT-qPCR analysis for the expression of the indicated mRNAs. GAPDH and malat1 were used as housekeeping reference mRNAs for the cytosolic and nuclear fractions, respectively. The ratio between the expression levels of a given RNA in each of the two fractions from G4C2-expressing cells was calculated and plotted considering the same ratio in control cells as 1. The SD was calculated from n = 3 independent experiments. * *p* < 0.05; ** *p* < 0.01.

Following the hypothesis that altered intracellular distribution of mRNAs might affect their function, we integrated the RNA-Seq data analysis using the Enrichr tool to identify specific pathways that might be significantly affected by *C9orf72* expression. As shown in Figure 3A, Reactome analysis shows that the pathways related to gene transcription are significantly enriched (adjusted *p*-value ≤ 0.05) in the nucleus-accumulated transcripts (“gene expression”, “RNA polymerase II transcription”, and “generic transcription”). Strikingly, 4 out of 10 of the most significant pathways that emerged from the analysis of the nuclear-enriched mRNAs include “membrane trafficking”, “Golgi-to-ER retrograde transport”, “vesicle-mediated transport”, and “Rab regulation of trafficking”, suggesting that several overlapping vesicle trafficking pathways might all be affected by the expanded *C9orf72* repeats.

Finally, to validate the transcriptomic data, we analyzed the expression of G4C2 RNA repeats in the nuclear and cytoplasmic fractions of transfected cells. As expected, G4C2 sequences are detectable almost exclusively in the nuclear fractions of the G4C2-transfected cells (Supplementary Figure S1), which is in line with the results of the FISH analysis and with the established concept that expanded *C9orf72* RNA repeats accumulate in cell nuclei. Furthermore, we selected six of the top ten mRNAs that are increased in the nuclei of G4C2 cells (Supplementary Table S2) and tested their differential localization via qPCR analysis. Among these, we included ArfGAP-1, a Golgi-localized protein that promotes the hydrolysis of GTP bound to the small guanine nucleotide-containing protein Arf1, and Vps54, a core subunit of the Golgi-associated retrograde protein (GARP) complex that is involved in retrograde transport from early and late endosomes to the trans-Golgi network (TGN) [25]. As shown in Figure 3B, we were able to confirm the differential localization of all the mRNAs selected from the RNA-Seq data. This indicates the reliability of the analysis performed and further suggests that Golgi-to-ER trafficking might be impaired by *C9orf72* expansion.

### 3.3. Expression of C9orf72 Expanded Repeats and DPRs Induces Golgi and ER Disorganization in Cell Lines

**Figure 4:**
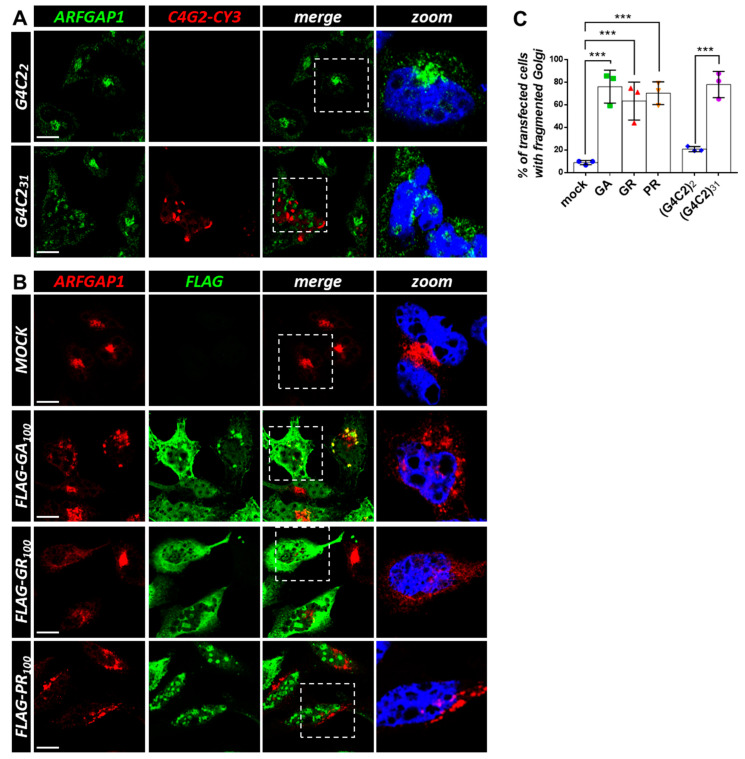
Both G4C2 expanded repeats and *C9orf72*-derived DPRs affect ArfGAP1 protein localization. (**A**) HeLa cells were transfected with plasmids coding for control (G4C2)_2_ and expanded (G4C2)_31_ repeats. After 24 h, the cells were subjected to fluorescence in situ hybridization (FISH) with a CY3-(C4G2)_4_ RNA probe (red) coupled with an immunofluorescence analysis using an anti-ArfGAP1 antibody (green). A merged image combining red and green signals is shown. Nuclei were stained with DAPI (blue), and representative magnifications of the highlighted areas (white dashed box) merged with the ARFGAP-1 signal are shown (zoom). Scale bar: 10 μm. (**B**) HeLa cells were transfected with an empty plasmid (mock) or plasmids coding for 100 dipeptides GR, PR, and GA *C9orf72*-derived DPRs, fused with a FLAG tag. After 24 h, thecells were subjected to an immunofluorescence analysis with anti-FLAG (green) and anti-ArfGAP1 (red) antibodies. A merged image combining red and green signals is shown. Nuclei were stained with DAPI (blue), and representative magnifications of the highlighted areas (white dashed box) merged with the ArfGAP-1 signal are shown (zoom). Scale bar: 10 μm. (**C**) The percentage of transfected cells with fragmented/dispersed ArfGAP-1-positive condensates is shown as the mean ± SD. At least 100 cells per condition in three independent experiments were scored. *** *p* < 0.001 via a one-way ANOVA versus the control cells is shown.

**Figure 5:**
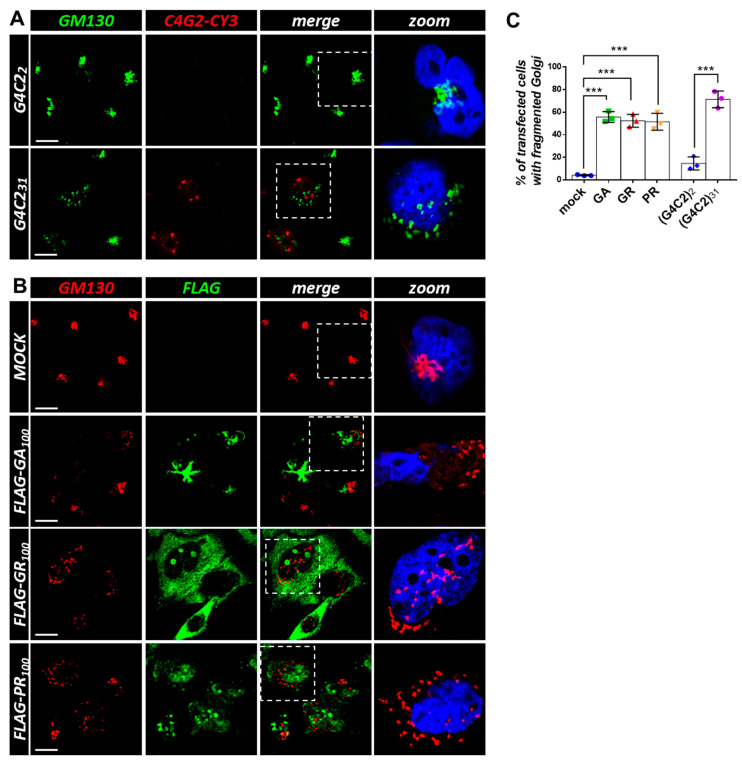
Both G4C2 expanded repeats and *C9orf72*-derived DPRs induce Golgi fragmentation. (**A**) HeLa cells were transfected with plasmids coding for control (G4C2)_2_ and expanded (G4C2)_31_ repeats. After 24 h, the cells were subjected to fluorescence in situ hybridization (FISH) with a CY3-(C4G2)_4_ RNA probe (red) coupled with an immunofluorescence analysis using an anti-GM130 antibody (green), a known Golgi marker. A merged image combining red and green signals is shown. Nuclei were stained with DAPI (blue), and representative magnifications of the highlighted areas (white dashed box) merged with the GM130 signal are shown (zoom). Scale bar: 10 μm. (**B**) HeLa cells were transfected with an empty plasmid (mock) or plasmids coding for FLAG-tagged C9orf72-derived DPRs. After 24 h, the cells were subjected to an immunofluorescence analysis with anti-FLAG (green) and anti-GM130 (red) antibodies. A merged image combining red and green signals is shown. Nuclei were stained with DAPI (blue), and representative magnifications of the highlighted areas (white dotted box) merged with the GM130 signal are shown (zoom). Scale bar: 10 μm. (**C**) The percentage of transfected cells with a fragmented Golgi apparatus is shown as the mean ± SD. At least 100 cells per condition in three independent experiments were scored. *** *p* < 0.001 via a one-way ANOVA versus the control cells is shown.

**Figure 6:**
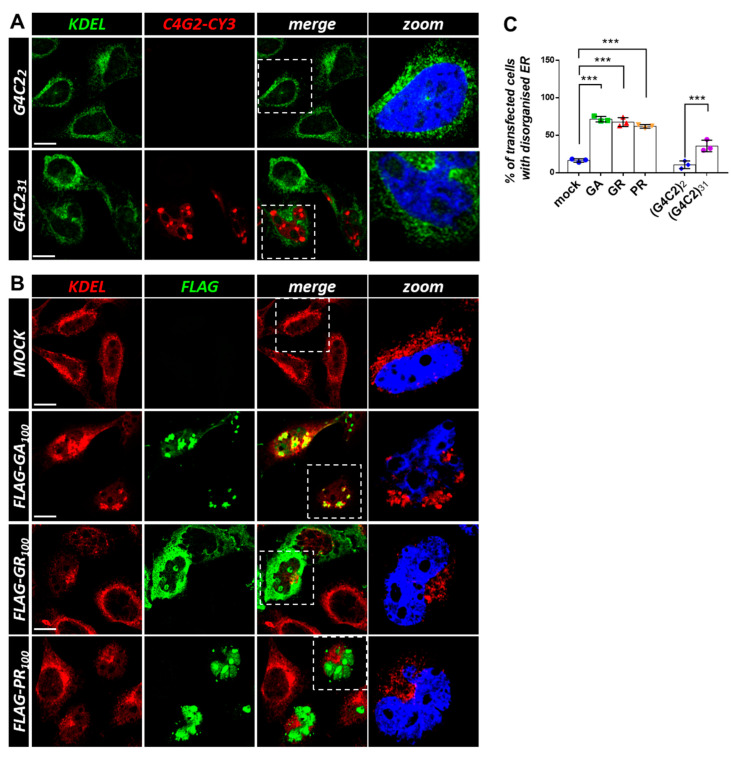
Both G4C2 expanded repeats and *C9orf72*-derived DPRs cause ER disorganization. (**A**) HeLa cells were transfected with plasmids coding for control (G4C2)_2_ and expanded (G4C2)_31_ repeats. After 24 h, the cells were subjected to fluorescence in situ hybridization (FISH) with a CY3-(C4G2)_4_ RNA probe (red) coupled with an immunofluorescence analysis using an anti-KDEL antibody (green), an epitope that characterizes ER-resident proteins. A merged image combining red and green signals is shown. Nuclei were stained with DAPI (blue), and representative magnifications of the highlighted areas (white dashed box) merged with the KDEL signal are shown (zoom). Scale bar: 10 μm. (**B**) HeLa cells were transfected with an empty plasmid (mock) or plasmids coding for FLAG-tagged C9orf72-derived DPRs. After 24 h, the cells were subjected to an immunofluorescence analysis with anti-FLAG (green) and anti-KDEL (red) antibodies. A merged image combining red and green signals is shown. Nuclei were stained with DAPI (blue), and representative magnifications of the highlighted areas (white dotted box) merged with the KDEL signal are shown (zoom). Scale bar: 10 μm. (**C**) The percentage of transfected cells with a disorganized ER is shown as the mean ± SD. At least 100 cells per condition in three independent experiments were scored. *** *p* < 0.001 via one-way ANOVA versus the control cells is shown.

To confirm that the vesicular trafficking between Golgi and ER is a target of expanded *C9orf72* repeat expression, HeLa cells were transfected with control and expanded (G4C2)_31_ repeats and subjected to an immunofluorescence analysis with an anti-ArfGAP-1 antibody coupled to a fluorescence in situ hybridization (FISH) analysis, using a Cy3-conjugated (C4G2)_4_ RNA probe. As shown in Figure 4A,C, in the presence of a control plasmid, ArfGAP-1 staining is concentrated in perinuclear tubular stacks in accordance with its known localization in the Golgi apparatus. On the contrary, in cells containing G4C2-positive nuclear foci, ArfGAP-1 staining is dispersed into smaller condensed structures throughout the cell. To verify whether the results obtained could be extended to *C9orf72*-derived DPRs, we transfected HeLa cells with plasmids expressing poly-GR, -PR, and -GA C9-DPRs, which are known to cause cell toxicity in a number of ALS model systems [26]. As shown in Figure 4B,C, the mock-transfected cells display perinuclear Golgi staining of ArfGAP-1. On the contrary, a significant fraction of cells expressing the transfected C9-DPRs show fragmented/dispersed ArfGAP-1-positive clusters, suggesting that C9-DPRs induce Golgi fragmentation similar to what is observed with the expanded *C9orf72* repeats. To verify this, an antibody anti-GM130, a protein located on the cis-surface of the Golgi apparatus that is commonly used as a Golgi marker, was used to stain the cells transfected with G4C2 and C9-DPRs. The results in Figure 5A–C show a clear pattern of Golgi fragmentation/disorganization in cells expressing the ALS-linked *C9orf72* constructs, which is consistent with what was observed with ArfGAP-1. Finally, a similar experimental setting was used to analyze the overall organization of the ER by using an antibody detecting KDEL epitope that characterizes ER-resident proteins and is used as an ER marker. As reported in Figure 6B,C, major effects are evident in cells expressing the C9-DPRs. Cells expressing poly-GA repeats specifically display condensed ER signals compared to the typical diffused perinuclear staining of the ER in the mock-transfected cells. Similarly, cells displaying G4C2 nuclear foci show signs of ER disorganization, though to a lesser extent (Figure 6A,C).

### 3.4. Functional Interaction between C9orf72 RNA Repeats and ArfGAP-1 in Drosophila

**Figure 7:**
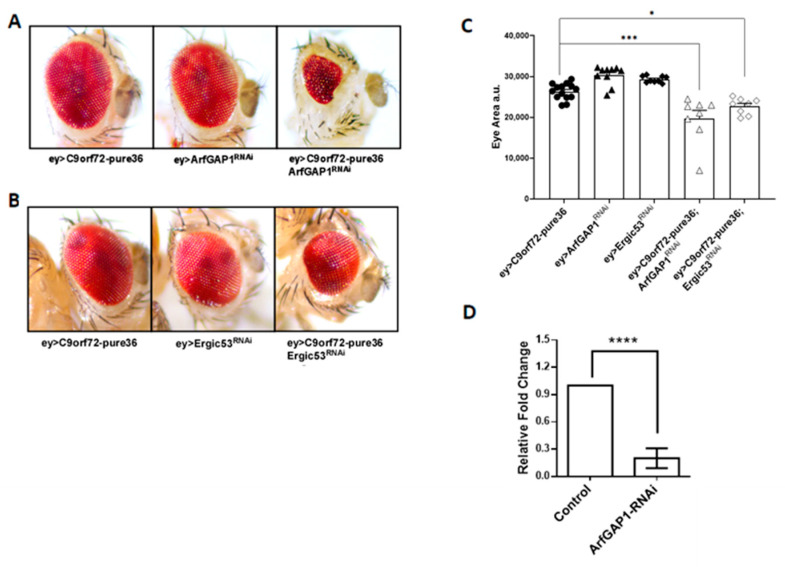
ArfGAP-1 RNAi-mediated knockdown exacerbates *C9orf72* RNA toxicity. (**A**) Representative images of Drosophila adult eyes from RNAi-expressing flies. No alteration in eye morphology is observed in female flies raised at 29 °C when 36 RNA *C9orf72* hexanucleotide repeats are expressed under the control of eyeless-GAL4. Similarly, the RNAi-mediated downregulation of ArfGAP-1 in female flies raised at 29 °C shows no effects. Conversely, when the expression of *C9orf72* RNA is combined with the repression of ArfGAP-1, a significant alteration in eye development is observed. (**B**) Analogously, the expression of *C9orf72* RNA in combination with the downregulation of ERGIC-53 generates relevant eye degeneration. (**C**) Statistical analysis of eye degeneration. The reduction in eye surfaces as an index of neurodegeneration was measured using a Fiji NIH Imagej, and the differences were tested using a one-way analysis of variance (ANOVA), followed by the Bonferroni multiple comparison test, and assessed using GraphPad Prism software, V. 8.1. * *p* < 0.05, *** *p* < 0.001. The experiment was repeated three times, and each dot on the graph represents a single fly eye. (**D**) A qPCR analysis of ArfGAP-1 mRNA levels in total RNAs extracted from the heads of flies expressing eyeless-GAL4 on its own or in combination with ArfGAP-1 targeting RNAi. **** *p* < 0.0001.

Building upon previous results, and to verify the functional implications of the alterations found, we used an in vivo model of *C9orf72*-ALS established in *Drosophila melanogaster* [27]. Thus, we employed the GAL4-UAS system to perform the RNAi-mediated downregulation of ArfGAP1 mRNA in fly tissues expressing *C9orf72* toxic species. The RNAi constructs were expressed early during eye development (eyeless-GAL4). The *Drosophila* compound eye contains around 700 units (ommatidia) which follow a precise and repetitive pattern of differentiation. Even small alterations in eye organization or the number of ommatidia are easily detected. Since major alterations to eye development usually do not interfere with fly viability, it is possible to study the expression of any *C9orf72* toxic species in this tissue [27]. Further, the Drosophila retina, which is composed of photoreceptors, neurons, and interneurons, is commonly used to study neurodegeneration [28]. As shown in Figure 7A,C, when we expressed 36 *C9orf72* RNA repeats in the eyes of female flies under the control of eyeless-GAL4, although raised at 29 °C to achieve the highest expression levels of this RNA, the flies showed no sign of neurodegeneration. Similarly, the expression of the RNAi construct able to downregulate *ArfGAP-1* mRNA, as confirmed via a qPCR analysis of RNAs extracted from the heads of flies (Figure 7D), did not cause any significant alteration in eye morphology. Most interestingly, when *C9orf72* RNA expression was combined with ArfGAP-1 downregulation, we observed striking eye degeneration (Figure 7A,C), supporting a strong genetic interaction between *C9orf72* RNA and ArfGAP-1. To confirm the functional link between Golgi-to-ER retrograde transport and *C9orf72* toxicity, we downregulated the expression of ERGIC-53, another membrane trafficking factor which affects endoplasmic reticulum–Golgi intermediate vesicles compartment (ERGIC) (Figure 7B). Again, while the RNAi-mediated downregulation of ERGIC-53 does not affect eye morphology on its own, it causes a visible reduction in eye size when it is combined with C9orf72 expression (Figure 7B,C).

### 3.5. Functional Relationship between C9orf72 DPRs and Golgi-to-ER Membrane Trafficking Proteins

**Figure 8:**
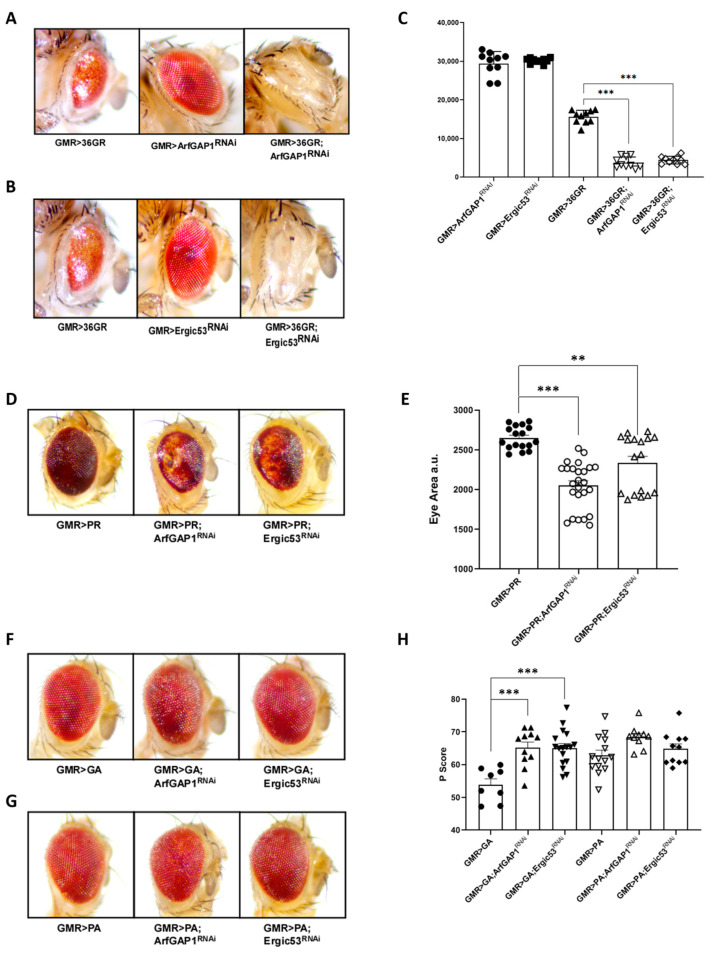
ArfGAP-1 downregulation worsens neurodegeneration due to *C9orf72* DPRs. We reduced either ArfGAP-1 or ERGIC-53 at 25 °C in flies expressing 36 repeats of several DPRs under the control of GMR-GAL4. (**A**) Thirty-six GR repeats cause visible retina degeneration which is even greater when combined with the downregulation of either ArfGAP-1 (**A**) or ERGIC-53 (**B**). (**C**) The reduction in eye surface was measured manually using a Fiji NIH Imagej, and the differences were tested using a one-way analysis of variance (ANOVA), followed by the Bonferroni multiple comparison test, and assessed using GraphPad Prism software, V. 8.1. *** *p* < 0.001. (**D**) A relevant worsening of retina degeneration is also observable in flies expressing 36 repeats of PR in combination with the downregulation of either ArfGAP-1 or ERGIC-53, which are associated with the prominent loss of pigmentation. (**E**) Eye surface reductions were measured manually by using a Fiji NIH Imagej, and the differences were tested via a one-way analysis of variance (ANOVA), followed by the Bonferroni multiple comparison test, and assessed via GraphPad Prism software, V. 8.1. ** *p* < 0.005, *** *p* < 0.001 (**F**) We utilized the open-source software Flynotyper to analyze the mild alterations in eye morphology due to the GA and PA DPRs. The Pscore represents Flynotyper’s output and summarizes several morphological parameters that increase with the worsening of the degeneration [29]. (**G**,**H**) This analysis shows that ArfGAP-1 or ERGIC-53 downregulation significantly exacerbates GA toxicity, though it does not affect PA toxicity. *p* score differences were tested via a one-way analysis of variance (ANOVA), followed by the Bonferroni multiple comparison test, and assessed using GraphPad Prism software, V. 8.1. *** *p* < 0.001. The experiment was repeated three times, and each dot in the graph represents a single fly eye.

To confirm that the effect of *C9orf72* RNA on the retrograde transport can be related to its ability to generate DPRs, we downregulated either ArfGAP-1 or ERGIC-53 in flies expressing 36 repeats of the GR dipeptide late during eye development under GMR-GAL4 at 25 °C [27], a condition that is sufficient to induce massive retina degeneration. Although 36 GR repeats cause strong degenerative phenotypes [27] (Figure 8A), they induce an even stronger reduction in eye surface that is associated with more severe neurodegeneration when combined with the downregulation of either ArfGAP-1 (Figure 8A,C) or ERGIC-53 (Figure 8B,C).

Finally, to evaluate the effect of ArfGAP-1 or ERGIC-53 on other DPRs, we downregulated either ArfGAP-1 or ERGIC-53 at 25 °C in flies expressing 36 repeats of PR, GA, or PA, all under GMR-GAL4 (Figure 8D–H) [27]. A relevant worsening of retina degeneration due to the downregulation of ArfGAP-1 or ERGIC-53 in flies expressing 36 repeats of DPR PR (Figure 8D,E) was evidenced. Also, in this case, the reduction in eye surface was measured as an index of neurodegeneration (Figure 8E). Since the GA and PA DPRs produce only slight alterations in eye morphology, we exploited Flynotyper, an open-source software that provides a reliable and quantitative method of automatically studying eye phenotypes [29]. The Flynotyper output, called the Pscore index, summarizes several morphological parameters and grows with the increase in morphology alterations [29]. A Pscore analysis showed that the downregulation of ArfGAP-1 or ERGIC-53 significantly exacerbates GA toxicity, though it does not cause any substantial effect on PA toxicity (Figure 8F–H).

## 4. Discussion

Robust evidence has been provided with respect to the impairment of nucleocytoplasmic (N/C) transport in *C9orf72*-ALS [5]. Both *C9orf72* RNA and dipeptide repeat proteins (DPRs) affect the N/C transport of RNAs and proteins in cellular and animal models of the disease [6,7,8,9,10,11,30]. In particular, we and others have demonstrated that the retention of RNAs in the nuclei of cells might play a role in the pathological process [7,8]. Nonetheless, how the nuclear retention of mRNAs influences MN degeneration remains unclear. Herein we have identified, via subcellular RNA fractionation and a sequencing analysis, 756 mRNAs that are differentially distributed in cells expressing *C9orf72* expanded repeats. Importantly, most of them (729 out of 756) are accumulated in the nuclei of *C9orf72* cells. Even more interestingly, pathway enrichment analysis of the nuclear-enriched mRNAs revealed that membrane trafficking is a major target of expanded *C9orf72* RNA repeats.

The mechanisms underlying these effects are currently unclear, although it is well established that the nuclear–cytoplasmic transport of specific mRNAs is strictly regulated in response to physiological and pathological conditions [31]. This is obtained via the interaction of cis-acting structural RNA motifs with N/C transport receptors, as well as through the combinatorial binding of RNA-binding proteins (RBPs) [32]. Considering that both *C9orf72* RNA repeats and DPRs directly bind and affect different N/C transport receptors and RBPs, it is tempting to speculate that *C9orf72* toxicity might arise from the mis-regulation of different N/C trafficking mediators, eventually leading to alterations in different classes of mRNAs. Yet further studies will be needed to verify whether this occurs in human *C9orf72*-ALS and to unveil the molecular mechanisms underpinning *C9orf72*-dependent alterations in the N/C transport of mRNAs. Indeed, a limitation of our study is that we used an in vitro cellular model, Hela cells overexpressing G4C2 repeats, that does not recapitulate the complex features of motor neuron degeneration in ALS. However, a recent study showed that human iPSC-derived motor neurons bearing TDP43 and VCP ALS mutations displayed clear N/C changes in the distribution of several mRNAs and proteins [33]. Interestingly, a significant overlap exists between the genes identified in this study and those described in our work (Supplementary Figure S2), an observation that might support the relevance of our findings.

Among the top mRNAs that are significantly affected in their nucleo-cytoplasmic expression in *C9orf72* repeat-transfected cells and those that were validated via qPCR analysis, ArfGAP-1 mRNA attracted our attention, given its central role in vesicular trafficking, particularly in Golgi-to-ER retrograde transport. ArfGAPs are indeed members of a protein family that promotes the hydrolysis of GTP bound to the small guanine nucleotide-containing protein ARFs[13,14]. ArfGAP-1 regulates Arf1, and both proteins control type-I coatomer (COPI) protein complex assembly, which contributes to the retrieval of proteins and lipids from the Golgi apparatus back to the ER. COPI protein complexes bind reversibly to membranes by deforming them into coated buds and concentrating protein cargos; both processes essential for protein sorting within the Golgi system [13,14]. This Arf1/ArfGAP-1 trafficking route has been extensively studied by several groups with cellular biological, biochemical, or pharmaceutical approaches, all of which are reconciled by a common model in which ArfGAP-1 regulates Arf1 and affects the uptake of cargo molecules in vesicles [13,14].

To complement our transcriptomics studies obtained in cultured transfected cells with more relevant systems and to provide functional evidence of a possible pathogenic role of ArfGAP-1 dysfunction in *C9orf72* toxicity, we took advantage of an in vivo model system of Drosophila neurodegeneration. Accordingly, we exploited in vivo Drosophila ALS models to study the effects of the RNAi-mediated downregulation of the *ArfGAP1* gene in flies expressing *C9orf72* RNA repeats or DPRs [27].

We found that *C9orf72*-mediated toxicity is affected by ArfGAP-1 and ERGIC-53, a different trafficking protein that works on the same pathway. The expression of 36 RNA *C9orf72* repeats, early on during eye development of the flies, did not significantly affect retina morphology. Similarly, no effect on retina morphology was observed with or the repression of either the *ArfGAP-1* or *ERGIC-53* genes. Conversely, the combination of *C9orf72* RNA repeats with ArfGAP-1 or ErgiC-53 repression unveiled strong alterations in eye development. Similarly, we observed a considerable exacerbation of retina degeneration in flies expressing *C9orf72* DPRs associated with ArfGAP-1 or ERGIC-53 downregulation. The expression of both *C9orf72* expanded RNA repeats and FLAG-tagged DPRs in cultured cell lines induces significant alterations in Golgi and ER morphology, which are linked to the effective mislocalization of endogenous ArfGAP-1 and might be a consequence of an alteration of its function. Indeed, ArfGAP-1 is spread far from the Golgi stacks in cells expressing DPRs or repeat expansion. Interestingly, the loss of function of ArfGAP-1 causes a dispersion of Golgi stacks similar to what we observe in *C9orf72* expressing cells [34]. This suggests that not only might *C9orf72* RNA repeats affect ArfGAP-1 expression through the mislocalization of its mRNA, but also that DPRs may directly impair ArfGAP-1 activity. A conclusion that is supported by the colocalization of at least some DPRs with ArfGAP-1 protein, which we occasionally observed in transfected cells.

Together, these observations support a model in which toxic species that are generated from mutant *C9orf72* (either RNA repeats or DPRs) directly affect the membrane trafficking processes regulated by ArfGAP-1 activity and contribute to ALS pathogenesis. A large number of genetic, morphologic, and mechanistic pieces of evidence indicate that defects in intracellular vesicle trafficking have a profound impact on motor neuron viability and activity, playing a pivotal role in ALS [35,36]. In particular, alterations in ER-to-Golgi communication have been described in genetic models of ALS associated with mutations in SOD1, FUS, TDP43, and VAPB, indicating that defects in this pathway might be critical in this disease [35,36]. Our study demonstrates that this conclusion can be extended to *C9orf72* mutations through the gain-of-function mechanisms exerted by novel RNA and/or protein species, and it is consistent with an early transcriptomic study in tissues from *C9orf72*-ALS patients which provided circumstantial evidence that *C9orf72* repeat expansions might affect Golgi-to-ER retrograde transport [37]. Recently, the tridimensional structure of the C9orf72 protein complex was resolved via cryo-EM, and it was demonstrated that the C9orf72-SMCR8 protein dimer behaves as a small GTPase-activating protein on the Arf family of GTPases, with a particular specificity for Arf1 [15,16]. Therefore, this structural and biochemical evidence strongly suggests that C9ORF72 haploinsufficiency, by directly affecting the GAP activity on Arf1, also significantly contributes to motor neuronal dysfunction causing ALS [15,16].

## 5. Conclusions

Overall, our observations suggest that the regulation of Golgi-to-ER trafficking by ArfGAP-1 might be a target of mutant *C9orf72* toxicity in ALS, and they support a multiple-hit model in which the gain-of-toxic effects exerted by *C9orf72* RNA repeats or DPRs on Arf1GAP-1 are added to the loss of GAP activity associated with C9orf72 haploinsufficiency, eventually converging on the same membrane trafficking pathway governed by Arf1.

## Data Availability

GEO accession number: GSE235448.

## Supplementary Materials

The following supporting information can be downloaded at: https://www.mdpi.com/article/10.3390/cells12152007/s1, Table S1: List of oligonucleotides used in qPCR experiments; Table S2: List of genes differentially distributed between the nucleus and cytosol in C9orf72 cells; Figure S1: G4C2 repeats accumulate in the nuclei of transfected HeLa cells; Figure S2: Comparison of mRNAs differentially distributed in the nucleus and the cytoplasm between G4C2 HeLa cells and ALS iPSC-derived motor neurons.
