# Supplementary material for: C9orf72 Toxic Species Affect ArfGAP-1 Function
Source: Cells. 2023 Aug 5;12(15):2007. doi: 10.3390/cells12152007 (PMC10416972; doi:10.3390/cells12152007)
Supplement: Supplementary file 1 [file cells-12-02007-s001.zip › Cells_FINAL_Supplementary Table S1_REVISED.pdf]

Supplementary Table S1. List of oligonucleotides used in qPCR experiments.

Gene name	Forward sequence	Reverse sequence
MALAT1	CCTGCAAATTGTTAACAGAAGGG	AGCTTCCGCTAAGATGCTAGCTT
GAPDH	TCTTTTGCGTCGCCAGCCGAG	TGACCAGGCGCCCAATACGAC
TPM4	CATCCAGCTCGTTGAGGAGG	TGGCCCGGTTTTCTATCACC
ARFGAP-1	CTCGGAGGGCCACAGTTATC	GCACTTCCAAAGGTCTCCCA
PDCD6	CACGGGTGTGTGGAAGTACA	GTGGAACTGGTCAGAGAGCC
MRPL4	ACCTGCACCCCGATGTTTTC	TGGTCTTGGCATAGCTAATTCTC
NUDT16	GGACAGCAGAGGAGCAGTG	GCATCTGCATCAGTATGGCG
VPS54	CGGGACCCGCAGTTGAGT	GGGTTCCTTGGGACACACAT
dARFGAP-1	AGGTTATGGCGGAAACGGAG	TGGAAAGCGTCGAGTCGAAA
pcDNA5	CGCAAATGGGCGGTAGGCGTG	CACTAAACGAGCTCGTCGACG
