# Supplementary material for: C9orf72 Toxic Species Affect ArfGAP-1 Function
Source: Cells. 2023 Aug 5;12(15):2007. doi: 10.3390/cells12152007 (PMC10416972; doi:10.3390/cells12152007)
Supplement: Supplementary file 1 [file cells-12-02007-s001.zip › Supplementary Figure S1.pdf]

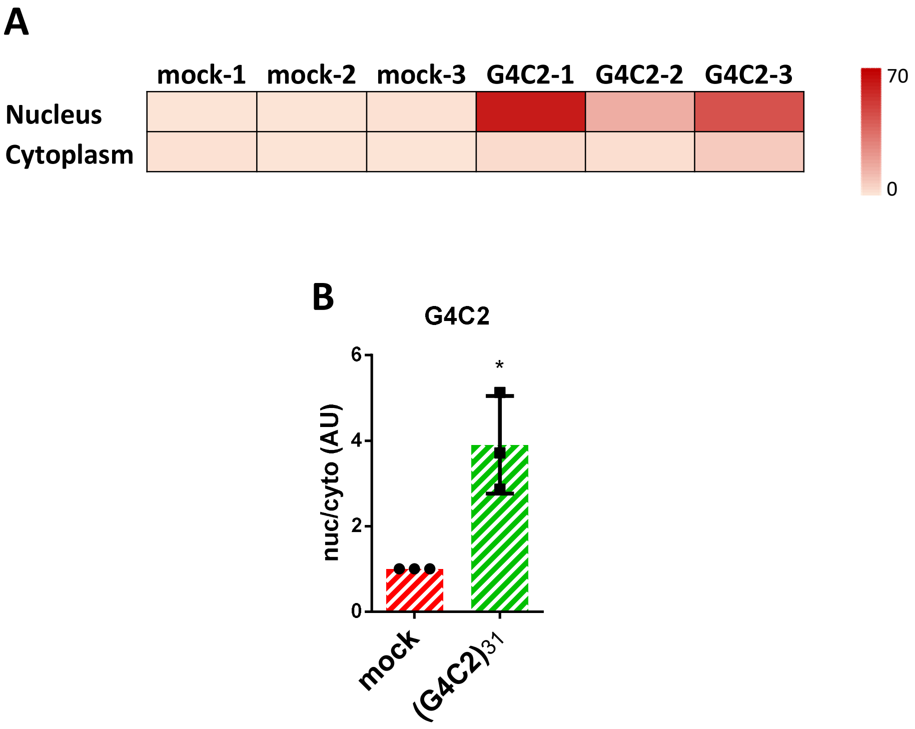

Supplementary Figure S1. G4C2 repeats accumulate in the nuclei of transfected HeLa cells. (**A**) Fastq files from the RNAseq analysis of nuclear and cytoplasmic fractions from mock- or $G4C2_{31}$- transfected HeLa cells were screened for the presence of G4C2 sequences. Heatmap of the reads found was produced with the number of reads proportional to the indicated color code. (**B**) HeLa cells were transfected with a control plasmid (mock) or a plasmid coding for $(G4C2)_{31}$. Total RNAs were extracted from purified nuclear and cytosolic fractions and analyzed by RT-qPCR for the expression of exogenous RNAs using primers annealing plasmid sequence upstream the cloning site of G4C2 repeat. *GAPDH* and *malat1* were used as housekeeping reference RNAs for the cytosolic and nuclear fraction, respectively. The ratio between the expression of a given RNA in each of the two fractions from G4C2-expressing cells was calculated and plotted considering the same ratio in control cells as 1. SD was calculated from n=3 independent experiments. *$p<0.05$.
