# Supplementary material for: C9orf72 Toxic Species Affect ArfGAP-1 Function
Source: Cells. 2023 Aug 5;12(15):2007. doi: 10.3390/cells12152007 (PMC10416972; doi:10.3390/cells12152007)
Supplement: Supplementary file 1 [file cells-12-02007-s001.zip › Supplementary Figure S2.pdf]

A

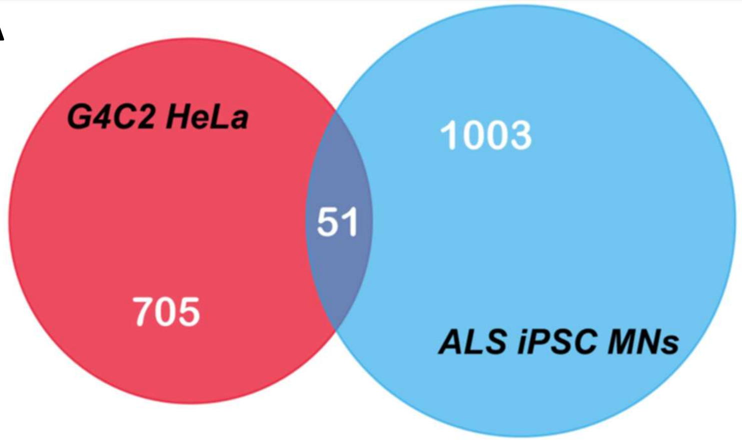

B

TPM4	*ZEB2*	*ANKEF1*
ZBTB34	*NAPB*	*RAB2A*
FGF2	*ZNF644*	*GTF2I*
CSNK1G3	*ECT2*	*CNOT8*
SETD2	*AKAP12*	*RAB11A*
FAM135A	*TRIM45*	*POLD2*
ZBTB10	*FYTTD1*	*NLN*
ZSCAN30	*MDM1*	*QDPR*
BNIP2	*RPLP0*	*PPP3CA*
AAK1	*NAA50*	*WDR41*
ZNF484	*RAB18*	*MSH2*
ZNF268	*TBC1D30*	*CYP20A1*
FGD5-AS1	*ARL4A*	*PTGES3*
DDHD1	*TMEM241*	*WDYHV1*
SEPHS1	*MLF1*	*ATP6V1D*
IBTK	*MTPAP*	*ABHD14A-ACY1*
MPP5	*DCLRE1B*	*PAICS*

Supplementary Figure S2. Comparison of mRNAs differentially distributed in the nucleus and the cytoplasm between G4C2 HeLa cells and ALS iPSC-derived motor neurons. Venn diagram showing the overlapping between RNAs differentially expressed in the nuclei and cytosol of HeLa cells overexpressing G4C2 repeats (our datasets) and those published by Ziff et al, combining datasets from *TARDBP* mutant, *VCPR155C* mutant, *VCPR191Q* mutant, and *VCPR191Q* knockin iPSC-derived motor neurons (**A**). $p<0.005$ (Hypergeometric test). The overlapping 51 genes are listed in (**B**).
