# Supplementary figures and images for: C9orf72 Toxic Species Affect ArfGAP-1 Function
Source: Cells. 2023 Aug 5;12(15):2007. doi: 10.3390/cells12152007 (PMC10416972; doi:10.3390/cells12152007)

# Experiment 1

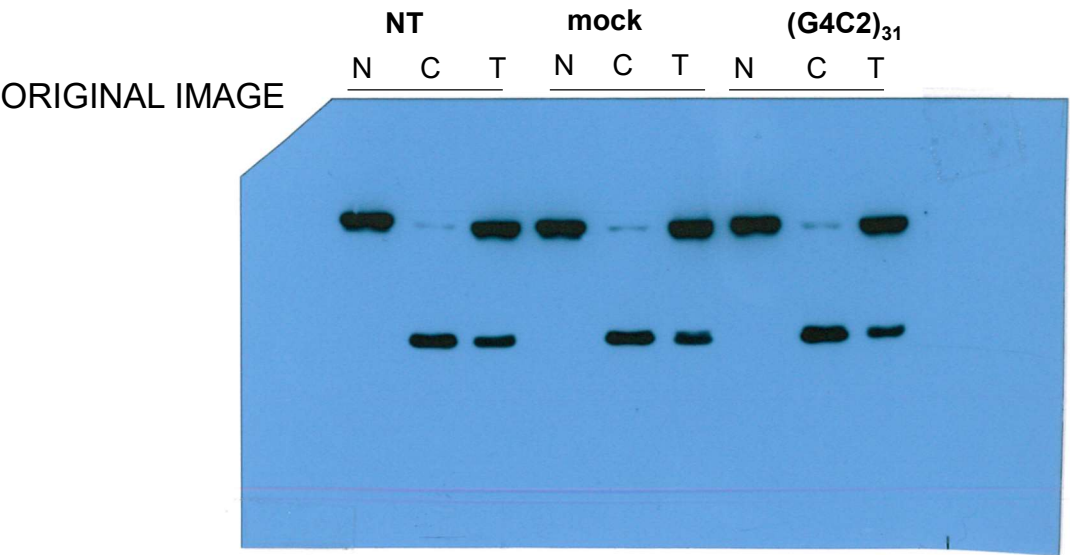

hnRNP H

GAPDH

FINAL IMAGE

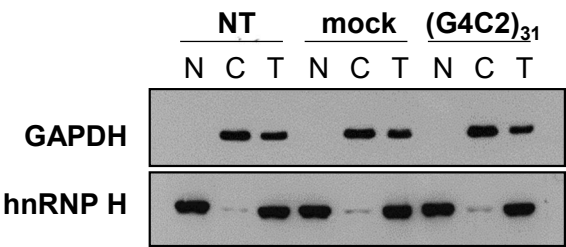

# Experiment 2

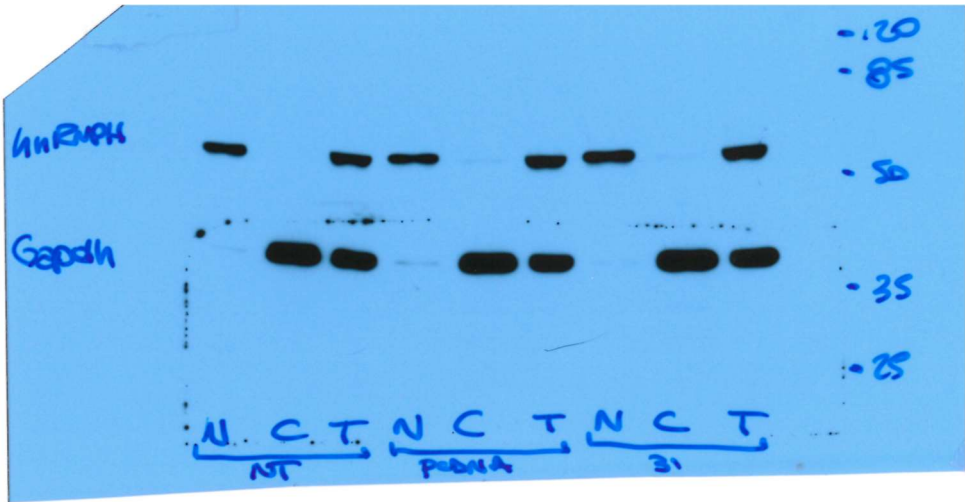

# Experiment 3

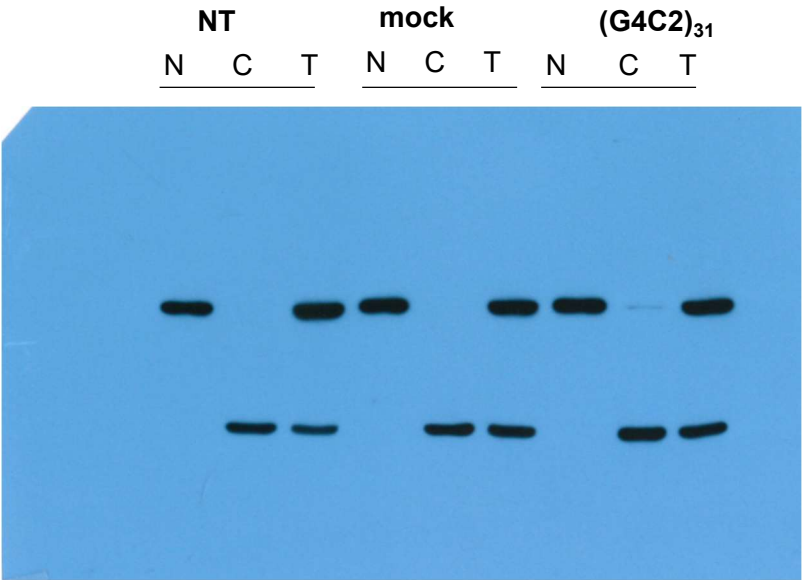

hnRNP H

GAPDH

Supplement: Supplementary file 1 [file cells-12-02007-s001.zip › original WB_REVISED.pdf]
